# Ameliorative effects of rutin against cisplatin-induced reproductive toxicity in male rats

**DOI:** 10.1186/s12894-018-0421-9

**Published:** 2018-11-21

**Authors:** Sarwat Jahan, Asma Munawar, Suhail Razak, Sara Anam, Qurat Ul Ain, Hizb Ullah, Tayyaba Afsar, Mahmoud Abulmeaty, Ali Almajwal

**Affiliations:** 1 Reproductive physiology laboratory, Department of animal sciences, Faculty of Biological Sciences, Quaid-i-Azam University, Islamabad, Pakistan; 2 Department of Biochemistry, Faculty of Biological SciencesQuaid-i-Azam University, Islamabad, Pakistan; 3 Department of Community Health Sciences, College of Applied Medical Sciences, King Saud University, Riyadh, Saudi Arabia

**Keywords:** Cisplatin, Rutin, Comet assay, Antioxidants, Testosterone, Histology

## Abstract

**Background:**

Cisplatin (CP) or cis-diammine dichloroplatinum (II) is a platinum based standard antineoplastic drug which is used against variety of solid tumors and neoplasms. The present study aimed to evaluate the shielding effects of rutin against CP induced testicular toxicity in rats.

**Methods:**

28 male rats were divided into four groups. First group was given saline orally while second group received intra-peritoneal (i.p) injection of cisplatin (7 mg/kg) on day first and received saline for next 13 days. Third group received i.p injection of cisplatin at day one and treated with rutin (75 mg/kg) orally for next 13 days. Fourth group was treated with rutin orally for 13 days. Animals were sacrificed on 14th day and reproductive organs were analyzed for various parameters.

**Results:**

Cisplatin treatment resulted in a significant decrease in daily sperm production, decrease in head length and % DNA in head, reduction of epithelial cell height, tubular diameter, reduction of the number of spermatogonia, spermatocytes and spermatids, increase in the thiobarbituric acid reactive substances (TBARS) and oxidative stress in testicular tissues, and change of the intra-testicular testosterone concentrations. Rutin co-treatment resulted in reversing cisplatin effect on DNA damage, sperm count, histological and biochemical parameters.

**Conclusion:**

These results indicated that rutin co-treatment could ameliorate cisplatin-induced reproductive toxicity in male rats.

## Background

Cisplatin (CP) or cis-diammine dichloroplatinum (II) is a platinum based standard antineoplastic drug which is used against variety of solid tumors and neoplasms. It is generally accepted that CP is a DNA alkylating agent that kills cells by several mechanisms including DNA damage, production of reactive oxygen species (ROS), and inducing apoptosis [1–3].

Use of CP for clinical purposes is limited by its side effects and has been reported toxic to reproductive system of male. It has been observed that male rats treated with CP, exhibit decline in reproductive organ weights and impaired fertility along with alterations in the growth and development of next generations [4]. Both endocrine and exocrine compartments are effected, resulting in impaired spermatogenesis, gonadal dysfunction, androgenesis [5, 6] and altering Leydig cell functions. It results in reduced sperm motility and sperm with normal morphology. Chromosomal abnormalities in spermatozoa and temporary or permanent azoospermia are associated side effects of CP treatment. These side effects are attributed to oxidative and nitrosative damage generated by CP [7]. Physiological and biochemical disturbance is caused by redox imbalance and increase in lipid peroxidation lead to germ cell apoptosis [8].

Flavonoids are potent antioxidants that inhibit lipid peroxidation and platelet aggregation [9, 10], protect the tissue from free radicals by direct scavenging ROS, reactive nitrogen species (RNS), and activating antioxidant enzymes [11]. Rutin is a flavone derivative which is composed of flavonol quercetin and disaccharide rutinose. Rutin is widely present in plants but is relatively rare in their edible parts. It was first discovered in nineteenth century in buckwheat which contain approximately 2–10% of total dry weight and 15% in young leaves. It is also found in apple, green tea, *Betula pendula* leaves, and other sources [12, 13]. The pharmacokinetics study of rutin (0.2080, 1.664 and 4.160 μg·ml^− 1^) in rat plasma showed that it is more stable and less degradable under different conditions [14]. Results of previous study conducted [15] and confirmed the uptake of rutin in small intestine of rat in free as well as in conjugated form. Its metabolites include 3-hydroxyphenylacetic acid, 3,4- dihydroxyphenylacetic acid, 3,4-dihydroxytoluene, homovanillic acid and 3,5, 7,30,50 pentahydroxyflavonol/quercetin [16]. About 9.2% of rutin was found in urinary excretions after oral administration of 50 mg/kg dose in rats [17]. Rutin is a strong antioxidant and it has many pharmacological benefits that include anti-tumor, anti-mutagenic, anti-diarrheal, anti-inflammatory, myocardial protecting, and act as immunomodulator as reviewed previously [18]. Rutin has antitumor activity by targeting different pathways including nuclear factor kappa-light-chain-enhancer of activated B cells (NFκB) and mitogen-activated protein kinase (MAPK) pathways, Interleukin-6/Signal transducer and activator of transcription 3 (IL-6/STAT3) pathway [19], apoptotic cell death (phospho-Bad, cleaved caspase 3 and cleaved Poly [ADP-ribose] polymerase (PARP) [20]. While, cisplatin involve c-Jun N-terminal kinases (JNK-mediated) apoptosis, inhibitor of apoptosis proteins (IAP) and c-FLIP_L_ degradation, Ripoptosome formation and autophagy-mediated apoptosis [21].

Several studies have suggested the protective effects of Rutin. It revealed renal protective effects on the reperfusion induced renal injury [22]. Previous studies have reported neuroprotective effects of rutin in rat model of ‘sporadic dementia of Alzheimer type’ [23] and in ‘dexamethasone-treated mice’ [24]. It is also known to improve endothelial functions by lowering nitric oxide (NO) production in human endothelial cells [13]. Protective effects of rutin against reproductive toxicity has also been confirmed by previous studies [25, 26]. Furthermore, it has inhibitory effects against generation of ROS and membrane lipid peroxidation. Previous studies reported that rutin detoxify the oxidative stress produced in the body by various drugs and chemicals e.g. gentamycin induced ototoxicity and nephrotoxicity and cyclophosophamide induced infertility [27, 28].

Keeping in view the protective effects of rutin, this study was designed to evaluate the protective effect of rutin against CP induced morphological and biochemical damage in the reproductive tissues.

## Methods

### Animals

A total of 28 adult male Sprague Dawley rats (300 ± 20 g) were purchased from the Rodent and Primates Facility of Animal Sciences Department, faculty of pharmacy. Rats were kept in clean cages and fed with standard laboratory food and water was available ad libitum*.* Animal house was maintained at temperature about 25 ± 5 °C and 12-h light/dark cycle. The experimental design and animal handling was assessed and approved by the ethical committee of Animal sciences department, QAU Islamabad.

### Experimental design

All animals included in the study are randomized and then divided into four treatment groups (*n* = 7/group). The investigators responsible for experimental procedure and data analysis were blinded and unaware of group allocation throughout the experiments. First group served as control group with intraperitoneal (i.p.) injection of normal saline at day one and oral saline treatment for next 13 days. Second group, CP injected group i.e. injected with CP (7 mg/kg) (Unistin, Korea United Pharm. Inc., Korea), at first day and then saline orally until the end of experiment. Third group of rats was injected with CP (7 mg/kg) at first day followed by daily oral dose of rutin (in the form of Rutin Trihydrate 75 mg/kg, MP Biomedicals, Inc., France) dissolved in normal saline (0.9% saline), throughout the experimental period. Fourth group received oral dose of rutin (75 mg/kg) per day for 13 days. The dose and rout of CP administration was according to Amin and Hamza. While the dose of rutin and time duration of experiment was based on previous studies [29, 30].

On 14th day, all the rats were weighed and euthanized by decapitatation; Trunk blood was obtained in heparinized tubes to be centrifuged for 15 min at 3000 rpm and then separated plasma was kept at -80 °C freezer until analysis. The testis and epididymis were dissected out, washed with normal saline and weighed. Right testicular tissue and epididymis were fixed in sera for histology while left testis was kept in liquid nitrogen at − 70° for antioxidant and left epididymis was minced for further processing of comet assay.

### Daily sperm production

Spermatids, resistant to homogenization (19th stage of spermatogenesis) in the homogenate, were counted by the method followed in Robb et al., 1978; shortly, frozen testis was thawed at room temperature, tunica albuginea was removed and then parenchyma was weighed. It was homogenized in 5 ml of a solution of NaCl 0.9% which included 0.5% triton X-100 followed by homogenization for 30 s. After 5-fold dilution, 20 μl of sample was placed into Neubauer chambers and number of late spermatids was counted under microscope at × 400 magnification. This value was used to get the total number of spermatids per testis. The number was used for determination of the number of spermatids per each gram (g) of testicular tissue which is the efficacy of sperm production. For the calculation of daily sperm production (DSP) the quantity of spermatids which were resistant to homogenization (per testis and per g of testis) was divided by 6.3 (DSP = Y/6.3).

### Assessment of DNA damage

DNA damage of each spermatozoon was measured via a modified neutral single cell electrophoresis (SCGE / comet assay) [31, 32]. Right epididymis was minced with forceps in phosphate buffer saline (PBS) in order to collect sperms and kept at 37 °C for comet assay. Slides were gently heated on slide warmer, covered with 100 μL of 1% regular melting point agarose (RMPA), covered with a large coverslip (22 × 50 mm) and put at 4 °C for at least 30 min until solidification of agarose. The coverslips were then removed and another layer of 85 μl low melting point agarose was spread above the first layer containing 20 μl of sperm suspension and 65 μl of 1% low melting point agarose (LMPA) at 37 °C. For cell lysing, the coverslip was removed and slides were placed in histology jar which contain a freshly prepared cold lysis buffer. The slides were incubated for 24 h at room temperature, washed with distilled water three times (20 min each) to remove detergent and salt traces. For conducting electrophoresis, slides were uniformly placed in columns in the neutral buffer containing electrophoresis tray. Electrophoresis was done for 20 min at 25 V. Slides were removed, covered with aluminum foil and air-dried overnight at 5 °C. The slides were rehydrated, stained with acridine orange and observed under epifluorescent microscopy (400X, Nikon AFX-1 Optiphot) and digital images were captured for subsequent analyses. Comet scoring was done by using TRITEK software. For analysis, 200 cells were counted from four fields of each slide counting the intact DNAs and the number of comets. Following sperm DNA comet parameters were recorded. Comet length (CL, μm), % DNA in head (%H), Tail DNA (TDNA, %) Tail moment (TM, μm), Tail length (TL, μm), and Olive moment (OL, μm) were included in this study.

### Histological analysis

Testis and epididymis were fixed in sera (4–6 h) and embedded in paraffin wax. Paraffin fixed tissues were mounted on wooden blocks and 6 μm thin sections were cut using Richert microtome (820 H, USA). Clean glass slides were albumenized and tissue ribbon were mounted onto slides and were placed at 62 °C overnight for the complete deparafinization. Slides were stained with hematoxylin and eosin in accordance with standard procedure and were examined under light microscope (Nikon, 187,842, Japan). Microphotography was done by Leica LB microscope (Germany) equipped with an automatic micro photographic system (Japan). Images were analyzed by using image J software.

### Biochemical analysis

Catalase activity (CAT) was assessed according to Chance and Maehly [33]. Superoxidase dismutase activity (SOD) was evaluated by recording colour intensity at 560 nm according to protocols of Kekkar et al. (1984) [34]. Results are expressed in units/mg protein. Peroxidase activity (POD) was assessed by the method of Chance and Maehly [33]. Glutathione reductase activity (GR) was determined by method of Carlberg et al. [35]. The assay for lipid peroxidation was carried out following the method of Wright et al. [36] which was modified by Iqbal et al. [37]. The amount of thiobarbituric acid reactive substances (TBARS) formed in each of the sample was assessed by measuring optical density of the supernatant at 535 nm using spectrophotometer against a reagent blank. The results were expressed as nM TBARS/min/mg tissue at 37 °C using molar extinction coefficient of 1.56 × 10^5^ /M cm.

### Hormonal analysis

Intra-testicular and plasma testosterone levels were measured by using Enzyme Linked Immuno Sorbant Assay (ELISA) kits. Intra-testicular testosterone was expressed as ng/g of tissue. The ELISA kit was purchased from Amgenix, Burlingame, CA, USA. Results were expressed as ng/ml.

### Statistical analysis

#### Sample size calculation

Sample size for current study was calculated by resource equation method [38] by using following formula:

$$ \mathrm{E}=\mathrm{Total}\ \mathrm{number}\ \mathrm{of}\ \mathrm{animals}-\mathrm{Total}\ \mathrm{number}\ \mathrm{of}\ \mathrm{groups} $$

Here, E is degree of freedom of analysis of variance (ANOVA). The value of E should lie between 10 and 20 to increase the chance of getting a more significant result. As, this method is based on ANOVA, it is applicable to all animal experiments [39].

In present study, we made four groups with seven animals each.

$$ \mathrm{E}=\left(7\times 4\right)-4 $$

$$ \mathrm{E}=28-4=24 $$

This sample size is adequate as chances of death of animals cannot be ignored.

All the data are shown as Mean ± SEM. One way analysis of variance (ANOVA) followed by tukey’s test was used for comparison of different groups using Graph pad prism 5 software. *P*-value less than 0.05 were set as a significant level.

## Results

### Daily sperm production (DSP)

A significant decrease (*p* < 0.01) in daily sperm production × 10^5^ and efficiency of daily sperm production × 10^5^ was observed in CP-treated group vs the control group. DSP (*p* < 0.01) and efficiency of sperm production (*p* < 0.05) was significantly increased in Cisplatin+Rutin treated group as compare to control. In rutin treated group, insignificant difference in DSP was observed when compared to control whereas a significant increase (*p* < 0.01) was seen when compared to co-treated groups (Table 1).

**Table 1:** Effect of Rutin treatment on Cisplatin induced alterations on production and efficiency of testicular daily sperm production

Groups (*n* = 4)	Daily Sperm Production × 10^5^ /testis	Efficiency of Sperm Production × 10^5^ /gram of testis
Control	12.26 ± 0.26	313.88 ± 6.79
Cisplatin	8.06 ± 0.39^a***^	177.10 ± 11.77^a**^
Cisplatin+Rutin	11.07 ± 0.25^b**^	264.87 ± 3.36^b*^
Rutin	12.06 ± 0.17	308.30 ± 10.93

Values are expressed as mean ± SEM,*p < 0.05, **p < 0.01, ***p < 0.001, ^a^ = Value vs control, ^b =^ Value vs cisplatin,^c^ = Value vs Cisplatin+Rutin

### Assessment of DNA damage

A highly significant rise in the mean value of number of comets/120 cells was observed in CP treated and Cisplatin+Rutin treated groups when compared with control group (*p* < 0.001 and *p* < 0.05 respectively). Whereas, rutin and CP treated groups showed significant reduction in number of comets when compared to co-treated (*p* < 0.05) group.

CP and co-treatment caused significant decrease in the mean value of head length when compared to control (*p* < 0.001, *p* < 0.05 respectively). However the mean value of tail length was significantly increased in CP treated (*p* < 0.001) group when it was compared to control group. A significant decrease in mean value of tail length was seen in co-treated (*p* < 0.05) group as compare to CP treated group.

**Fig. 1:**
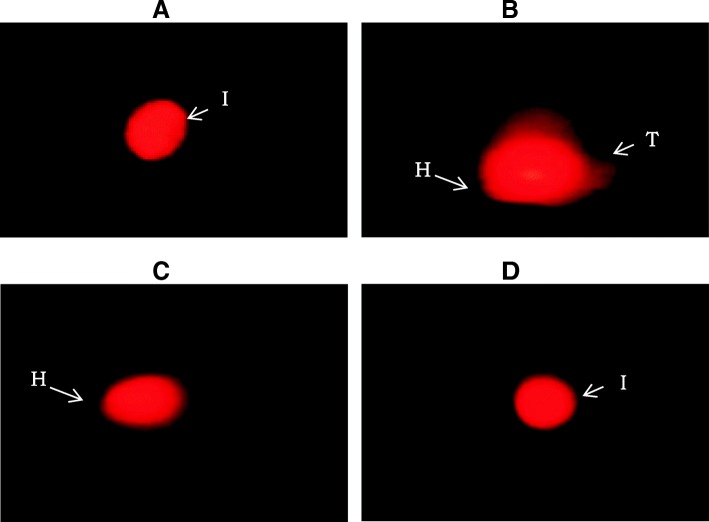
Total length of chromatin dispersion in the sperm treated with (**a**) control, (**b**) Cisplatin treated, (**c**) Cisplatin+Rutin treated, (**d**) Rutin alone treated groups. 40 X. I: Intact, T: Tail, H: Head

The mean value of % DNA in head exhibited significant decrease in CP treated (*p* < 0.001) and co-treated (*p* < 0.05) groups when compared with control group. Rutin treatment caused no significant difference in % DNA in head as compared to co-treated group. The mean values of percentage DNA in tail and tail moment showed a significant increase in CP treated group (*p* < 0.001) and co-treated (*p* < 0.01) groups when compared to control. In co-treated group, significant increase (*p* < 0.05) in tail moment was observed when compared to CP treated group. (Table 2, Fig. 1).

**Table 2:** Effect of Rutin treatment against cisplatin induced DNA damage in adult male rats

Groups (*n* = 4)	Control	Cisplatin	Cisplatin + Rutin	Rutin
No.of Comets/120 Cells	27 ± 2.97	58 ± 3.91^a***^	43 ± 1.10^ab*^	29 ± 1.41^c*^
Head length (μm)	158.71 ± 6.43	119.80 ± 3.87^a***^	132.29 ± 2.37^a*^	153.19 ± 7.42
Tail length (μm)	30.64 ± 1.91	53.25 ± 3.79^a***^	40.25 ± 1.27^b*^	33.74 ± 2.47^c*^
DNA in head (%)	88.80 ± 1.59	71.52 ± 2.87^a***^	74.37 ± 3.61^a*^	84.22 ± 1.77
DNA in tail (%)	10.84 ± 3.20	24.57 ± 1.88^a***^	22.51 ± 2.18^a*^	12.65 ± 1.12^c*^
Tail moment (μm)	3.60 ± 0.52	11.79 ± 1.59^a***^	6.95 ± 1.76^ab*^	3.84 ± 0.46^c*^

Values are expressed as mean ± SEM *p < 0.05, **p < 0.01, ***p < 0.001, ^a^ = Value vs control, ^b^ = Value vs cisplatin, ^c^ = Value vs Cisplatin+Rutin

### Histomorphometric analysis

**Fig. 2:**
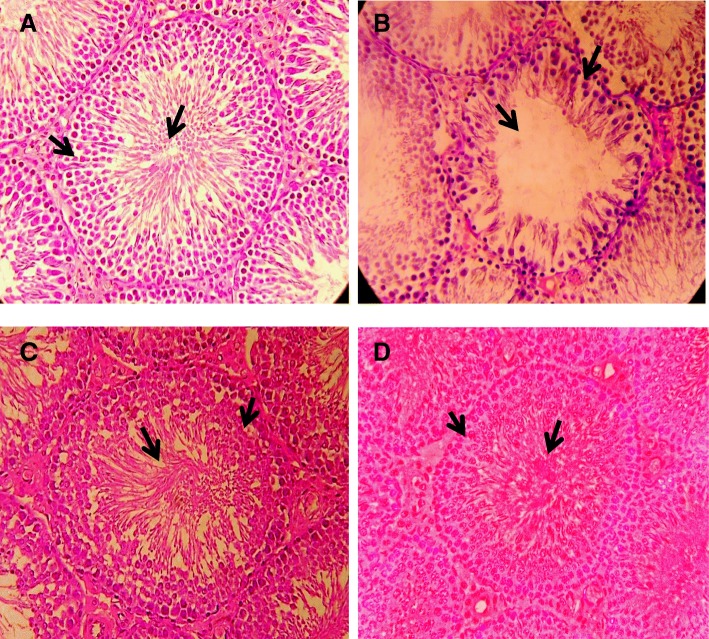
Photomicrograph of seminiferous tubules of adult male rat testis (H&E, 40X) from: (**a**) Control group showing normal morphology of seminiferous tubule with thick germinal epithelium containing proliferating germ cells (arrow) and lumen filled with spermatids (arrow); (**b**) Cisplatin treated group showing disruption in spermatogenesis, increased tubular lumen (arrow) and sloughing of germinal epithelium (arrow); (**c**) Cisplatin+Rutin treated group showing decreased tubular diameter (arrow) and decreased interstitial space (arrow) as compared to treated group; (**d**) Rutin treated group showing slight increase in lumen (arrow) and interstitial spaces (arrow) as compared with control group

Histomorphological examination of rat’s testis of control group showed normal spermatogenesis along with thick stratified germinal epithelium containing proliferating germ cells and narrow lumen filled with spermatozoa (Fig. 2a). In CP treated rats, noticeable disruption in normal spermatogenesis along with considerable degeneration of seminiferous tubules, reduction in the tunica albuginea thickness and tubular diameter, increased interstitial space and sloughing of germinal epithelium was observed (Fig. 2b). Testicular section from Cisplatin+Rutin treated rats and Rutin treated group did not showed any major differences in seminiferous tubules when compared to control group. Normal spermatogenesis along with lumen filled with mature spermatids was observed as compared to CP treated group (Fig. 2c & d).

A highly significant reduction (*p* < 0.001) in the seminiferous tubule diameter and germinal epithelial thickness was seen in CP treated group as compared to the control group. Cisplatin+Rutin treatment caused significant increase (*p* < 0.01) in tubular diameter and epithelial height when compared to CP treated. No significant change was observed between rutin and co-treated groups (Table 3).

**Table 3:** Morphometrical evaluation of testicular tissue in different treatment

Groups (*n* = 4)	Interstitial space (μm)	Tunica albugenia height (μm)	Seminiferous tubule diameter (μm)	Seminiferous tubule epithelial height (μm)	Tubular lumen (μm)
Control	2.41 ± 0.05	30.55 ± 0.37	248.39 ± 3.04	98.50 ± 0.92	22.03 ± 0.38
Cisplatin	11.86 ± 0.11^a***^	15.96 ± 0.53^a***^	215.03 ± 2.72^a***^	50.59 ± 0.61^a***^	51.42 ± 1.4^a***^
Cisplatin + Rutin	5.75 ± 0.14^b*^	22 ± 0.53	238.56 ± 3.97^b**^	67.52 ± 0.62^ab***^	25.69 ± 1.65^b**^
Rutin	4.28 ± 0.09	28.49 ± 0.42	240 ± 2.93	76.6 ± 0.7^a**^	29.6 ± 0.91

Values are expressed as mean ± SEM, *p < 0.05, **p < 0.01, ***p < 0.001, ^a^ = Value vs control,^b^ = Value vs cisplatin

Mean tubular lumen diameter and interstitial spaces between seminiferous tubules in CP treated group showed significant increase (*p* < 0.001) vs control group. Cisplatin+Rutin treatment significantly restored values of tubular lumen diameter (*p* < 0.01) and interstitial space (*p* < 0.05) significantly as compared to CP treated group. However, non-significant change was observed among Rutin treated and co-treated groups (Table 3).

A highly significant reduction (*p* < 0.001) was recorded in tunica albuginea height in CP group as compared to control. However, Cisplatin+Rutin treatment caused non-significant change in tunica albugenia height as compared to Cp treated group (Table 3).

A significant reduction (*p* < 0.001) in the spermatogonia, primary spermatocytes, secondary spermatocytes and spermatids was observed in CP treated group as compared to control group. Cisplatin+Rutin treatment treatment significantly restored germ cell number as compared to CP treated groups (*p* < 0.001, *p* < 0.01, *p* < 0.05 respectively) (Table 4).

**Table 4:** Effect of Rutin treatment against cisplatin induced alterations on germ cell stages in adult male rats

Groups (*n* = 4)	Spermatogonia	Primary Spermatocytes	Secondary Spermatocytes	Spermatids
Control	61.85 ± 1.14	45.05 ± 1.09	43.55 ± 1.01	91.25 ± 1.07
Cisplatin	47.8 ± 1.14^a***^	34.35 ± 1.04^a***^	29.9 ± 1.09^a***^	68 ± 1.57^a***^
Cisplatin+Rutin	55.85 ± 0.79^b***^	39.1 ± 0.83^ab**^	37.75 ± 0.81^a***b*^	73.75 ± 1.63^a***^
Rutin	56.3 ± 1.40	40.3 ± 0.87^a**^	35.3 ± 1.00	78.7 ± 2.19^a***^

Values are expressed as mean ± SEM,*p < 0.05, **p < 0.01, ***p < 0.001, ^a^ = Value vs control, ^b^ = Value vs cisplatin, ^c^ = Value vs Cisplatin+Rutin

### Biochemical analysis

Catalase (CAT) and Superoxide dismutase (SOD) activity showed significant reduction (*p* < 0.01) in CP treated group as compared to control group. However, CAT activity was significantly increased (*p* < 0.05) when Cisplatin+Rutin treated group was compared to CP treated group. Similarly, rutin treated group have a significant increase in SOD activity when compared co-treated group (*p* < 0.05). The concentration of peroxidase (POD) was significantly decreased in CP treated (*p* < 0.001) and co-treated (*p* < 0.05) groups when compared to control. Co-treatment produced a significantly high (*p* < 0.01) activity of POD than CP groups. The activity of glutathione (GR) was significantly reduced in CP treated (*p* < 0.01) group and co-treated group (*p* < 0.05) when compared to control. Rutin treatment significantly enhanced the concentration of GR as compared to Co-treated (*p* < 0.05) group. A significantly high level of TBARS was observed in CP treated group and co-treated group as compared to control group (*p* < 0.001). While Co-treated group showed significant decrease (*p* < 0.01) in TBARS when compared to CP treated group (Table 5).

**Table 5:** Effect of rutin on Antioxidant enzyme status and oxidative stress marker level in Cisplatin treated rats testis

Groups (*n* = 4)	CAT (U/mg protein)	SOD (U/mg protein)	POD (nmole/mg protein)	GSR (nM NADPH oxidized/min/mg tissue)	TBARS (nM/mg tissue)
Control	31.04 ± 2.96	29.75 ± 0.61	10.00 ± 1.33	28.21 ± 0.84	2.58 ± 0.75
Cisplatin	17.20 ± 1.91^a**^	17.90 ± 0.61^a**^	1.98 ± 0.25^a***^	14.60 ± 0.67^a**^	10.27 ± 0.74^a***^
Cisplatin + Rutin	28.23 ± 1.79^b*^	19.19 ± 1.48^a*^	6.68 ± 0.69^a*b**^	18.86 ± 1.30^a*^	7.46 ± 0.77^ab**^
Rutin	23.15 ± 0.87	29.36 ± 3.48^c*^	7.82 ± 0.98	28.09 ± 2.67^c*^	3.06 ± 0.50^c**^

Values are expressed as mean ± SEM *p < 0.05, **p < 0.01, ***p < 0.001, ^a^ = Value vs control, ^b^ = Value vs cisplatin, ^c^ = Value vs Cisplatin+Rutin

### Hormonal analysis

There was no significant difference in plasma testosterone concentration in CP treated, Cisplatin+Rutin treated and rutin treated groups as compared to the control animals as well as when compared with each other (Table 6). A significant reduction (*p* < 0.01) in intra-testicular testosterone concentration was seen in CP treated group vs control group. Cisplatin+Rutin treatment caused significant increase in intra-testicular testosterone concentration as compared to CP treated group (*p* < 0.01 respectively) while no significant change was seen when comparison was made with Rutin treated group (Table 6).

**Table 6:** Testicular tissue (ng/g tissue) and plasma (ng/ml) testosterone concentrations in different groups

Groups (*n* = 4)	Testicular T levels (ng/g tissue)	Plasma T levels (ng/mL)
Control	6.80 ± 5.12	1.23 ± 0.34
Cisplatin	1.65 ± 0.79^a**^	1.69 ± 0.63
Cisplatin+Rutin	8.93 ± 1.59^b***^	1.65 ± 0.49
Rutin	6.84 ± 0.61	1.62 ± 0.54

Values are expressed as mean ± SEM, *p < 0.05, **p < 0.01, ***p < 0.0001, ^a^ = Value vs control, ^b^ = Value vs treated, ^c^ = Value vs Cisplatin+Rutin

## Discussion

In the present study, protective effects of rutin were investigated against the toxic effects induced by CP in reproductive tissues of adult male rats. Treatment with CP indicated marked alterations in testicular histopathology and antioxidant enzyme status. Histopathological alterations include reduction in seminiferous tubules diameter and epithelial height while increase in tubular lumen. On the other hand, increased oxidative stress in the testicular tissues was observed in groups treated with CP. CP has been extensively studied for its beneficial effects in destruction of cancerous cells. Besides its beneficial effects, it has been reported toxic to the reproductive tissues in male [40]. CP exerts its effects by increasing the level of Reactive Oxygen Species (ROS) thus reducing antioxidant enzymes status leading to alteration in testicular machinery [41]. Co-treatment with rutin protected the testicular tissues against detrimental effects of CP and reduced the oxidative stress in the tissue. Rutin has been reported as a strong antioxidant having antitumor, anti-inflammatory and cytoprotective effects [23, 42, 43].

In present study, significant reduction in daily sperm production (DSP) was noted in CP treated group as compared to control while rutin treatment in combination with CP restored sperm production. These results indicate that treatment with rutin can shield reduction in DSP within the testis by ameliorating the adverse effects of CP. Previously, it was reported that CP treatment impaired spermatogenesis [4], caused decrease in number and motility of spermatozoa and increase in number of sperm with abnormal morphology, chromosomal abnormalities in spermatozoa, and temporary or permanent azoospermia [44]. These effects of CP on sperm have been linked to the oxidative stress inducing potentials of the compound. CP induced ROS generation in the testis and sperm and caused cell death in the seminiferous epithelium [45]. ROS are the reactive oxygen and nitrogen species which are generated in the mitochondria during normal cellular activities. However, excessive ROS can lead to alteration in the mitochondrial membrane which in turn leads into more production of ROS [46]. This excessive ROS generation can cause oxidation of lipids, proteins and DNA, which can lead into poor semen parameters of the individual [47]. In present study CP treatment not only cause degeneration of epithelial cells but also led into reduction in DSP. Cotreatment with rutin exhibited ameliorative effects on sperm parameters and induced DSP in the animals treated with CP. These results seem to be because of the antioxidant potentials of rutin [48].

CP treatment caused increase in the number of comets, % DNA in tail, tail lengths and tail moment while % DNA in head and head length was reduced in CP treated group as compared to control group. However, rutin co-treatment showed significant reduction in DNA damage caused by CP. In previous studies, it was reported that CP induced DNA damage, chromosomal abnormalities in spermatozoa and inhibited DNA synthesis [49]. Similarly, increase in tail length in the treated groups is in accordance with previous findings [50]. It was described in earlier studies, that rutin protected mitomycin C induced DNA damage [51]. These protective effects might be due to the reduction in the oxidative stress in the testicular tissues that might have protected the sperm DNA in the exposed groups.

The results of histological examination indicated that CP administration for 13 days caused a significant decrease in seminiferous tubular diameter, seminiferous tubules epithelial height, tunica albuginea height, increase in tubular lumen, interstitial space and reduction in germ cells number and deceleration in spermatogenesis. These findings were consistent with the previous studies that showed that seminiferous tubules contain arrested spermatogenic cells at various stages of division. Similarly, reduced mean seminiferous tubule diameter and degenerative changes in germinal cell layer thickness were prominent in the group that was treated with CP. Another effect of CP was the distortion of seminiferous tubule epithelium in terms of sloughing [52]. Previously, CP-induced testicular toxicity was also evidenced by the histopathological lesions [53]. Similar studies also indicated that CP adversely affected the testicular tissues and remarkably decreases the production of sperm by increasing oxidative stress [54]. In our study, rutin treatment reversed the toxic effects of cysplatin on spermatogenesis. Previously Abarikwu and his colleagues found that rutin caused decrease in Cyclophosphamide induced oxidative stress and testicular damage by sustaining antioxidant level in epididymis and testis [25]. Similarly, rutin ameliorated CP induced nephrotoxicity in male rats by reducing oxidative stress [55]. In another study, rutin was known to attenuate CP induced renal inflammation and apoptosis by inhibiting nuclear factor, −light chain enhancer of B cells (NFκB) and tumor necrosis factor α (TNF-α) pathway involved in inflammation, caspase-3 mediated-tubular cell apoptosis [56]. These findings suggest that CP induce inflammation and reduce apoptosis, while rutin acts as anti- inflammatory.

In present study, there was a significant decrease in sperm count as well as a significant reduction in spermatogenesis was observed that is similar to earlier finding [57]. Previously, it has been reported that there was a significant decrease in sperm count, spermatogonia and primary spermatocyte of male mice treated with 1 mg/kg and 2 mg/kg of CP as compared with the control group, while there is a significant decrease in spermatid in the mice who were treated with 2 mg/kg only, this indicates the cytotoxicity of CP on all types of germ cells spermatogenesis [52]. The decrease in germinal epithelium thickness and seminiferous tubular diameter might be because of the inhibition of spermatogenesis, which must also contribute to reduce the sperm count. These damaging and harmful effects were amended by rutin administration. These results are similar to the earlier findings in which rutin administration reversed the reduction in spermatogenesis and steroidogenesis against cisplatin and cyclophosphomide [25]. In another study, rutin treatment (5 mg and 10 mg) has remarkably and dose-dependently enhanced the sperm count as well as percentages of viable and motile spermatozoa when compared to control group [58].

The results of our study showed that CP reduced plasma antioxidant levels and caused failure of antioxidant defense mechanism which increases free radical damages in body [59], inducing apoptosis [60] and stimulating inflammation. In our experiment level of Catalase (CAT) was significantly reduced in CP treated groups as compared to control animals. These findings are in accordance with previous findings in which CP treatment caused reduction in CAT levels [61–63]. Similarly, significant decrease in the level of SOD, POD and GR activity was observed in CP treated group that are in accordance with previous studies in which CP treatment caused reduction in the levels of antioxidant enzymes SOD and POD are important in cell defense mechanism because SOD converts the superoxide ions to elemental oxygen and hydrogen peroxide, while POD converts hydroden peroxide into water via oxidation-reduction reaction. Rutin treatment caused a significant increase in the level of SOD and POD [64]. Hence reduced levels of SOD and POD caused an increase in the levels of ROS and RNS leading to lipid peroxidation and oxidative damage. Previous studies also reported the protective effect of rutin on testicular injury that may be caused by scavenging ROS and increasing SOD and CAT activities. The decreased level of glutathione activities in this study is consistent with many studies reported earlier [64]. Lipid peroxidation can lead to cell damage which is irreversible [65]. In present study CP treatment caused an increase in the level of TBARS, in both CP treated groups which is in accordance with previous literature in which single i.p injection at the dose of 7 mg/kg disturbed antioxidant level [46]. Rutin administration in combination with CP lowered the concentration of TBARS. These results are similar to previous literature in which rutin treatment caused significant reduction in TBARS level that was elevated by treatment with potassium bromate [66]. It directs ameliorative effects of rutin on cysplatin induced oxidative damage.

CP administration in the present study caused no significant change in plasma testosterone concentration, but significant decrease in intra-testicular testosterone concentration. Previously, it was observed that mice which were administered with 1 and 2 mg/kg of CP, showed a significant decrease in testosterone levels as compared with control group [52]. 3β- Hydroxysteroid dehydrogenas (3β-HSD) and 17β- Hydroxysteroid dehydrogenas (17β-HSD) are main enzymes in testicular androgenesis and play a key regulatory role in testicular steroidogenic events. Low level of these enzymes by cisplatin treatment might be the cause of decreased testosterone concentration. Rutin treatment normalized the hormonal level in our study, that is similar to previous studies in which rutin coadministration prevented cyclophosphamide induced decrease in spermatogenesis and steroidogenesis [25].

## Conclusion

In conclusion, the findings of the present study demonstrate that oxidative stress caused by free radicals plays an important role in the development of CP-induced testicular damage and sperm count. Moreover, it has shown that rutin, because of antioxidant property, partially reverses some of the CP-related pathological effects on testicular tissue. Therefore, it is suggested that rutin may be used as a potent therapeutic agent combined with CP to reduce its side effects. However, further work on safety in higher animal models and eventual clinical trials are required prior to making any definitive conclusions regarding the potential utility of the drug on humans.

## Abbreviations

17β-HSD: 17β- Hydroxysteroid dehydrogenas
3β-HSD: 3β- Hydroxysteroid dehydrogenase
ANOVA: One way analysis of variance
CAT: Catalase activity
CP: Cisplatin
DSP: Daily sperm production
ELISA: Enzyme Linked Immuno Sorbant Assay
GR: Glutathione reductase
IAP: Inhibitor of apoptosis proteins
IL-6/STAT3: Interleukin-6/Signal transducer and activator of transcription 3
JNK-mediated): c-Jun N-terminal kinases
LMPA: Low melting point agarose
MAPK: Mitogen-activated protein kinase
NFκB: Nuclear factor kappa-light-chain-enhancer of activated B cells
NO: Nitric oxide
PARP: Poly [ADP-ribose] polymerase
POD: Peroxidase
RMPA: Regular melting point agarose
RNS: Reactive nitrogen species
ROS: Reactive oxygen species
SOD: Superoxidase dismutase
SSGE: Single cell gel electrophoresis
TBARS: Thiobarbituric acid reactive substances
TNF-α: Tumor necrosis factor α
